# Anagliptin promotes apoptosis in mouse colon carcinoma cells via MCT-4/lactate-mediated intracellular acidosis

**DOI:** 10.3892/etm.2022.11211

**Published:** 2022-02-15

**Authors:** Qi Li, Xiaoling Qin, Xiaotong Kou, Jingyu Li, Zhongsha Li, Chang Chen

**Affiliations:** 1 The Biotherapy Center, Harbin Medical University Cancer Hospital, Harbin, Heilongjiang 150086, P.R. China; 2 Department of Pharmacology (State-Province Key Laboratories of Biomedicine-Pharmaceutics of China, Key Laboratory of Cardiovascular Research, Ministry of Education), College of Pharmacy, Harbin Medical University, Harbin, Heilongjiang 150086, P.R. China

**Keywords:** anagliptin, monocarboxylate transporter-4, apoptosis, lactate, pH gradient

## Abstract

Cancer cells frequently exhibit an acidic extracellular microenvironment, where inversion of the transmembrane pH gradient is associated with tumor proliferation and metastasis. To elucidate a new therapeutic target against cancer, the current study aimed to determine the mechanism by which the dipeptidyl peptidase-4 inhibitor anagliptin regulates the cellular pH gradient and concomitant extracellular acidosis during cancer progression. A total of 5x10^5^ CT-26 cells (resuspended in phosphate buffer saline) were injected subcutaneously in the right flank of male BALB/c mice (weighing 25-28 g). The tumor samples were harvested, and lactate was detected using a lactate assay kit. Immunohistochemistry was used to detect the Ki67 and PCNA. MTT assay and flow cytometric were used to detect cell viability. Intracellular pH was detected by fluorescence pH indicator. The results revealed that anagliptin effectively reduced tumor growth, but did not affect the body weight of treated mice. Anagliptin reduced the accumulation of lactate in tumor sample. Treatment with anagliptin stimulated the apoptosis of CT-26 cells. And lactate excretion inhibition is accompanied by an increase in extracellular pH (pHe) after treatment with anagliptin. Furthermore, anagliptin induced intracellular acidification and reversed the low pHe gradient via monocarboxylate transporter-4 (MCT-4)-mediated lactate excretion. Additionally, anagliptin reversed the aberrant transmembrane extracellular/intracellular pH gradient by suppressing MCT-4-mediated lactate excretion, while also reducing mitochondrial membrane potential and inducing apoptosis. These data revealed a novel function of anagliptin in regulating lactate excretion from cancer cells, suggesting that anagliptin may be used as a potential treatment for cancer.

## Introduction

Cancer is a leading cause of death worldwide (1). Despite the development of various drugs for cancer therapy, numerous anticancer agents offer little therapeutic benefit. This, together with their associated adverse effects, limit their clinical outcomes (2). A reversed extracellular/intracellular pH gradient is associated with tumor growth and metastasis (3). These phenotypes have been ascribed, mechanistically, to effects of extracellular acidosis on several processes (4). Disrupting extracellular/intracellular pH gradient by inhibiting membrane transporters may be a therapeutic strategy (5). In addition, inhibiting these transporters induces toxic intracellular acidosis (6); therefore, maintaining an alkaline intracellular environment is necessary for cancer cell survival (7).

Lactate is a bioenergetic metabolite formed in the absence (fermentation) or presence of oxygen and is used by cells as an oxidative substrate (8). Lactate, in addition to being an energy substrate, is a gluconeogenic and signaling factor in multiple cell types (9). Cancer cells produce high levels of intracellular lactate, inducing an increase in lactate extrusion to compensate for cytosolic acidity, which causes the cytosol to become alkalinized (10). However, inefficient lactate release caused by the functional disruption of monocarboxylate transporters (MCTs) decreases intracellular pH (pHi) and slows tumor growth (11). This suggests that targeting MCTs may represent a new strategy for anticancer treatment.

Dipeptidyl-peptidase-4 (DPP-4) is a ubiquitously expressed transmembrane exopeptidase found on the surface of numerous hematopoietic cells (12). DPP-4 has sparked scientific interest over the last 10 years, with numerous studies describing its role in tumor immunology and the prognosis of patients with cancer (13-16). Various DPP-4 inhibitors are used to treat type II diabetes with an absence of serious side effects (17). However, it remains unclear whether DPP-4 inhibitors are beneficial or detrimental to existing tumors. In the present study, animal and cell experiments were conducted to verify whether anagliptin could inhibit cancer cells growth through MCT-4 signaling pathway. In addition, the suppressive mechanism of anagliptin was further explored.

## Materials and methods

### Cell culture

Murine colon carcinoma CT-26 cells (obtained from American Type Culture Collection) were maintained in RPMI-1640 medium (HyClone; Cytiva) supplemented with 10% fetal bovine serum (Gibco; Thermo Fisher Scientific, Inc.), 100 U/ml penicillin and 100 mg/ml streptomycin (Gibco; Thermo Fisher Scientific, Inc.). CT-26 cells were cultured at 37˚C in 5% CO_2_.

### Experimental animals

A total of 45 healthy male BALB/c mice (weight, 25-28 g; age, 6-8 weeks) were obtained from the Animal Center of The Second Affiliated Hospital of Harbin Medical University (Harbin, China; license no. SCXK2019-001). Mice were maintained in groups of five animals per cage under a 12-h light/dark cycle under controlled conditions (23±1˚C and 55±5% humidity). Autoclaved water and food were available *ad libitum* to mice. The experimental protocol was designed in accordance with Institutional Laboratory Animal Care and Use Committee standards. All animal-involving experimental procedures performed in the present study were in accordance with and approved by the Institutional Animal Care and Use Committee of Harbin Medical University Cancer Hospital (Harbin, China; approval no. KY2016-16).

### Animal models

The 45 healthy male BALB/c mice were randomly assigned to the following experimental groups: i) Model group (n=15), ii) anagliptin group (n=15) and 5-fluorouracil (5-Fu) group (n=15). A total of 5x10^5^ CT-26 cells (re-suspended in phosphate buffer saline) were injected subcutaneously in the right flank. All mice were weighed daily. Tumor growth was monitored by palpation, and the onset when tumors were detectable was noted. If no visible nodules were observed at the site of injection within 2 weeks, it was considered that this tumor sample could not form a tumor nodule. The tumor nodule was measured every day after appearance. Individual tumor volumes were measured with calipers and calculated using the following formula: Volume=[π/6 x (width)^2^ x length]. After visible nodules were observed, the murine cancer model was treated with the DPP-4 inhibitor anagliptin (20 mg/kg/day, MedChemExpress) daily by oral administration (the usage and dosage of anagliptin was determined based on previous experiments, Li *et al*, unpublished data). The present experiments were treated with 5-fluorouracil (5-Fu,25 mg/kg/day, Selleck Chemicals) every other day intraperitoneally as a positive control (the usage and dosage of 5-Fu was determined based on previous experiments, Li *et al*, unpublished data) (18). The murine cancer model was treated with vehicle (saline). As soon as the volume of the subcutaneous tumor reached 3,000 mm^3^, mice were euthanized using CO_2_ inhalation in their home cages. The CO_2_ flow rate was 30-40% of the chamber volume per min as recommended by the Canadian Council on Animal Care guidelines on euthanasia of animals used in science (19). Subsequently, cervical dislocation followed to ensure death. Samples of solid tumors were harvested, and then stored at -80˚C. Three independent experiments were performed.

### Immunohistochemistry (IHC)

Tissues were fixed in 4% paraformaldehyde for 30 min at 4˚C, embedded in paraffin and then four sections (5-µm) were cut at multiple levels. Tissues were dewaxed with xylene for 15 min at room temperature, rehydrated with decreasing concentrations of ethanol (absolute ethanol, 2 min; 95% ethanol, 2 min; 85% ethanol, 2 min; 75% ethanol, 2 min) and washed with tap water at room temperature. Antigen retrieval was performed in 10 mM citrate buffer (pH 6.0) for 10 min at 100˚C. Tissue sections were cooled, blocked for endogenous peroxidase with 3% H_2_O_2_ at room temperature for 15 min and blocked for endogenous biotin with an avidin-biotin kit (Biocare Medical, LLC) at room temperature for 15 min according to the manufacturer's protocol. Tissue sections were incubated at room temperature with 10% goat serum (cat. no. WGAR1009-5; Wuhan Servicebio Technology Co., Ltd.) for 30 min, then incubated at room temperature for 1 h with primary antibodies for Ki67 (1:200; cat. no. WL01384a) and proliferating cell nuclear antigen (PCNA; 1:200; cat. no. WL03213) (both from Wanleibio Co., Ltd.). Primary-antibody binding was detected by biotinylated species-specific secondary antibody (1:200; cat. no. A0277; Beyotime Institute of Biotechnology) at 37˚C for 30 min, followed by a horseradish peroxidase conjugate (Vectastain Elite ABC kit; Vector Laboratories, Inc.) according to the manufacturer's instructions. Immunoreactivity was revealed with 3,3'-diaminobenzidine (cat. no. G1212; Wuhan Servicebio Technology Co., Ltd.). Sections were counterstained with hematoxylin (cat. no. G1004; Wuhan Servicebio Technology Co., Ltd.) at room temperature for 3 min. Sections were examined microscopically with an optical microscope (Olympus Corporation), and images were determined using digital microscopy with SPOT Advanced software v5.3 (SPOT Imaging; Diagnostic Instruments, Inc.).

### Measurement of cell viability

CT-26 cells were seeded into 96-well plates (5x10^5^ cells/well) and incubated at 37˚C for 24 h. Upon reaching 90% confluence, the cells were treated with different concentrations of anagliptin (0.125-4 mM) at 37˚C for 24 h with or without serum. Subsequently, 20 µl MTT (pH 4.7) was added to each well and the cells were incubated at 37˚C for another 4 h. Then, 100 µl 10% sodium dodecyl sulfate (SDS) 0.01 M HCl was added and the cells were incubated at 37˚C overnight to dissolve the formazan crystals. Absorbance was measured at 570 nm.

### Flow cytometric assay

After treatment with anagliptin for 24 h, CT-26 cells were harvested (centrifuged at 4˚C, 825 x g for 10 min) and re-suspended at a density of 1x10^4^ cells/ml in 1X Annexin binding buffer (dilute 5X annexin-binding buffer 1:4 with deionized water; cat. no. V13246; Invitrogen; Thermo Fisher Scientific, Inc.). After double staining with FITC-Annexin V and propidium iodide using the FITC Annexin V Apoptosis Detection kit (cat. no. BB-4101; BestBio) according to the manufacturer's protocol, cells were analyzed using a FACScan^®^ flow cytometer equipped with Cell Quest software (version 5.1; BD Biosciences) according to the manufacturer's protocol to detect early and late apoptosis of cells. All experiments were performed in triplicate.

### Cell transfection

For small interfering (si)RNA transfection, CT-26 cells were plated at 3x10^5^ cells/ml in OPTI-MEM serum-reduced medium (cat. no. 31985-062; Gibco; Thermo Fisher Scientific, Inc.), and transfected with 100 pmol of targeted siRNA or NC siRNA using 5 µl of Lipofectamine^®^ RNAiMAX Reagent Agent (cat. no. 13778-075; Invitrogen; Thermo Fisher Scientific, Inc.) for 48 h in CO_2_ incubator at 37˚C according to the manufacturer's protocol. Mouse MCT-4 siRNA (cat. no. sc-40120) and control siRNA (cat. no. sc-37007) were purchased from Santa Cruz Biotechnology, Inc. After transfection of MCT-4 for 48 h, the cells were harvested, then the subsequence assay was performed.

### Lactate concentration

The concentration of lactate in culture media was detected using a commercial lactic acid kit (cat. no. A019-2-1; Nanjing Jiancheng Bioengineering Institute) according to the manufacturer's protocol.

### Measurement of extracellular pH (pHe)

Briefly, the culture medium was harvested from each group. Experiments were conducted in room atmosphere, at 37˚C. Extracellular pH of the media was verified using pH meter (cat. no. ECPHWP60001, Thermo Fisher Scientific, Inc.).

### Measurement of pHi

pHi was detected using a fluorescence pH indicator [2',7'-bis(carboxyethyl)-5,6-carboxyfluorescein; BCECF] according to the manufacturer's protocol (cat. no. BB-48121; BestBio). Briefly, stock solutions (1 mM) of BCECF were made by dissolving in DMSO before use. CT-26 cells (5x10^4^ cells in 24 well) were washed, then stained with 5 µM (final concentration) of the cytoplasmic pH-sensitive dye BCECF in HEPES-buffer for 20 min at 37˚C in the dark. The fluorophores were loaded into the cells through passive diffusion (to avoid compromising cell membrane integrity) (20). For the fluorescence measurements, the following wavelengths were set: Excitation at 492 and 438 nm; emission at 525 nm. Fluorescence levels were measured using a fluorescence microscope (LSM800; Carl Zeiss AG).

### JC-1 staining

According to the manufacturer's instructions, a total of 2.5 µM JC-1 (cat. no. M8650; Beijing Solarbio Science & Technology Co., Ltd.) was added to the media of CT-26 cells (5x10^4^ cells in 24 well) for 10 min at 37˚C. Cells were then washed in HBSS media (136 mM NaCl, 3 mM KCl, 1.25 mM CaCl_2_, 1.25 mM MgSO_4_, 10 mM HEPES and 2 mM D-glucose), Monomeric JC-1 green fluorescence mission and aggregate JC-1 red fluorescence emission were measured using a fluorescence microscope at 530/590 nm (IX73; Olympus Corporation).

### Western blot analysis

Frozen tissue was immersed in 600 µl lysis buffer (containing 40% SDS, 60% RIPA (cat. no. G2002; Wanleibio Co., Ltd.) and 1% protease inhibitor (cat. no. 539131; MilliporeSigma) and was centrifuged at 4˚C 17,400 x g for 30 min. The supernatant was collected and stored at -80˚C. CT-26 cells were washed with ice-cold PBS and centrifuged at 825 x g for 10 min at 4˚C. Subsequently, 70 µl lysis buffer containing 1% protease inhibitor solution was added. The cell suspension was pipetted for 30 min on ice, then centrifuged at 17,400 g for 30 min at 4˚C. The supernatant was collected and stored at -80˚C. The protein concentration was determined with the BCA Protein Assay kit (Bio-Rad Laboratories, Inc.). The samples (100 µg per lane) were separated by SDS-PAGE on 10% gels, then the separated proteins were transferred to a nitrocellulose membrane. The membrane was blocked in 5% non-fat milk overnight at 4˚C. Then, it was incubated with the following primary antibodies against: Bcl-2 (cat. no. WL01556), Bax (cat. no. WL01637), caspase-3 (cat. no. WL04004), cleaved-caspase-3 (cat. no. WL01992), cytochrome *c* (cyto C; cat. no. WL02410), MCT-4 (cat. no. 22787-1-AP) and GAPDH (cat. no. WL01114). Antibodies against Bcl-2, Bax, caspase-3, cleaved-caspase-3, cyto C and GAPDH were purchased from Wanleibio, Co., Ltd. Antibodies against MCT-4 were purchased from ProteinTech Group, Inc. All antibodies were diluted to 1:200 in PBS. After washing with PBS-0.1% Tween-20, membranes were incubated with fluorescence-conjugated goat anti-rabbit IgG secondary antibody (1:10,000; cat. no. 926-32211; LI-COR Biosciences) at room temperature for 1 h. Western blot bands were captured on the Odyssey Infrared Imaging System (LI-COR Biosciences) and quantified using Odyssey v3.0 software (LI-COR Biosciences) by measuring the densitometry for each group.

### Statistical analysis

Obtained data were expressed as the mean ± standard deviation. Three independent experiments were performed. Data were assessed with SPSS 22.0 software (IBM Corp.). One-way analysis of variance followed by Bonferroni's correction as post hoc test was used for multiple comparisons. P<0.05 was considered to indicate a statistically signiﬁcant difference.

## Results

### Anagliptin reduces tumor growth

**Figure 1:**
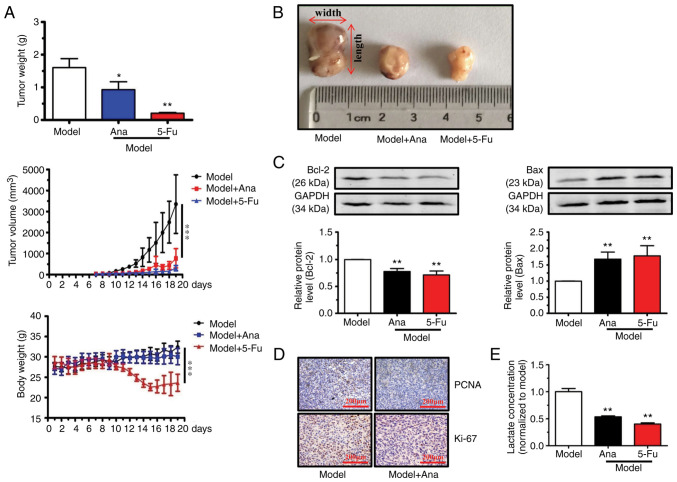
Treatment with Ana suppresses tumor growth in a mouse xenograft model. (A) Body and tumor weights were measured and tumor volumes were calculated as volume=π/6 x (width)^2^ x length. (B) Images of the xenograft tumors in BALB/c mice. (C) Bcl-2 and Bax protein expression levels were assessed by western blotting following treatment with Ana. (D) Immunohistochemical analysis of Ki67 and PCNA in a mouse xenograft model (scale bars, 200 µm). (E) Ana down-regulated the lactate concentration in axenograft model. Data are presented as the mean ± SD. The results are representative of three independent experiments performed in triplicate. ^*^P<0.05, ^**^P<0.01 and ^***^P<0.001 vs. the model group. Ana, anagliptin; PCNA, proliferating cell nuclear antigen; 5-Fu, fluorouracil.

On day 7, tumor samples grew into visible nodules. After which the growth of tumor nodule in BALB/c mice grew rapidly to >3,000 mm^3^ in size and the mice were euthanized using CO_2_ and sacrificed in model group (Fig. 1A). Animal models were treated with anagliptin (20 mg/kg/day, by oral administration) and 5-Fu (25 mg/kg/day, intraperitoneally) once tumor nodules appeared (day 7). Our pre-experiments confirmed that this dosage of anagliptin (20 mg/kg/day) was well tolerated, as no weight loss or other signs of toxicity were observed in normal mice (Li *et al*, unpublished data). In the animal experiment, 5-Fu (25 mg/kg/day, intraperitoneally) was used as the positive control. And our pre-experiments also indicated that the usage and dosage of 5-Fu was also tolerated (unpublished data). As revealed in Fig. 1A, tumor nodule growth slowed from day 7 today 10. The tumor nodule growth rapidly from day 11 to the end of the experiment (day 19) in mice after treatment with anagliptin and 5-Fu. After harvesting and measuring the tumor samples at the end point of experiments, treatment with anagliptin and 5-Fu was observed to significantly decrease the tumor volume compared with model group (Fig. 1A and B). Furthermore, anagliptin administration did not influence body weight (Fig. 1A), but treatment with5-Fu decreased the body weight from day 10 to the end of experiments.

Western blot analysis revealed that anagliptin treatment promoted Bax and decreased Bcl-2 expression levels (Fig. 1C). The expression levels of Ki67 and PCNA in the animal model were next examined with IHC. A markedly higher Ki67 and PCNA positive signal was observed in the model group, while treatment with anagliptin caused a marked decrease in the expression of Ki67 and PCNA (Fig. 1D). Furthermore, treatment with anagliptin down-regulated the concentration of lactate in animal models (Fig. 1E). These results indicated that treatment with anagliptin had the ability to suppress the growth of tumor.

### Anagliptin induces apoptosis in CT-26 cells

**Figure 2:**
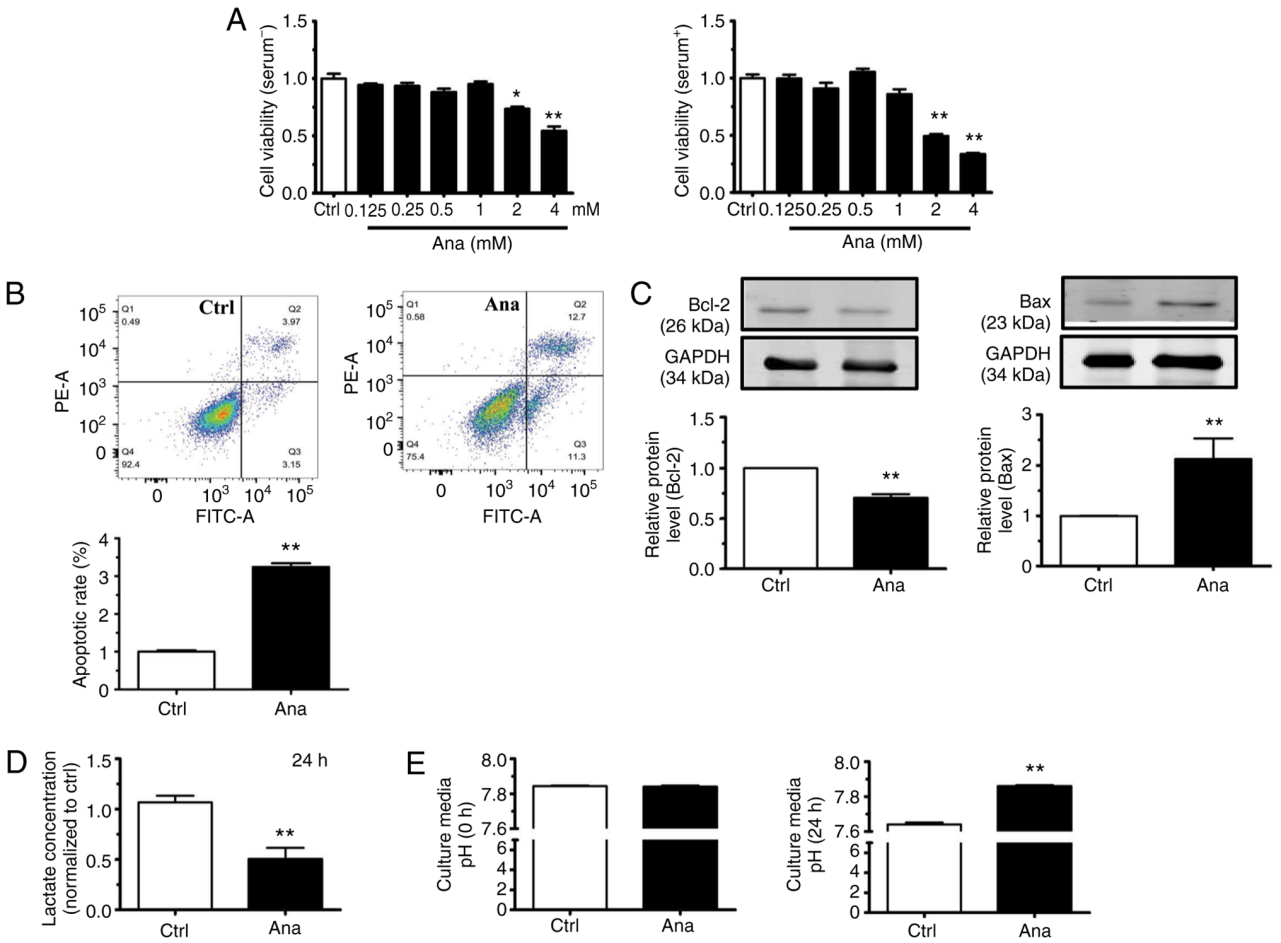
Ana promotes CT-26 cell apoptosis. (A) Ana (>2 mM) decreased the cell viability of CT-26 cells. (B) Flow cytometric analysis revealed that Ana induced CT-26 cell apoptosis. The right lower quadrant indicates early apoptotic cells; the right upper quadrant indicates late apoptotic cells and the left upper quadrant indicates necrotic cells. (C) Western blot analysis of Bcl-2 and Bax protein expression levels after treatment with Ana. (D) Ana suppressed lactate excretion in CT-26 cells after 24 h. (E) Ana reversed the low extracellular pH in CT-26 cells. The results are representative of three independent experiments performed in triplicate. Data are presented as the mean ± SD. ^*^P<0.05 and ^**^P<0.01 vs. Ctrl group. Ctrl, Control; Ana, anagliptin.

Anagliptin, at concentrations ≥2 mM, decreased the cell viability of CT-26 cells after culturing for 24 h with or without serum (Fig. 2A). Therefore, 2 mM of anagliptin was then used in subsequent studies. Flow cytometric analysis revealed that the proportion of late apoptotic CT-26 cells was significantly increased following treatment with anagliptin compared with in the control group (Fig. 2B). Anagliptin treatment also significantly reduced Bcl-2 expression levels and increased Bax expression levels (Fig. 2C). Those results indicated that treatment with anagliptin stimulated the apoptosis of CT-26 cells.

In addition, anagliptin-treated CT-26 cells produced lower levels of lactate in the cell culture medium (Fig. 2D). Meanwhile, anagliptin reversed low extracellular pH (pHe) in cultured CT-26 cell medium after 24 h (Fig. 2E). The results demonstrated that, after treating with anagliptin in CT-26 cells, the excretion of lactate was decreased which accompany with the high extracellular pH.

### Anagliptin suppresses MCT-4-mediated lactate excretion

**Figure 3:**
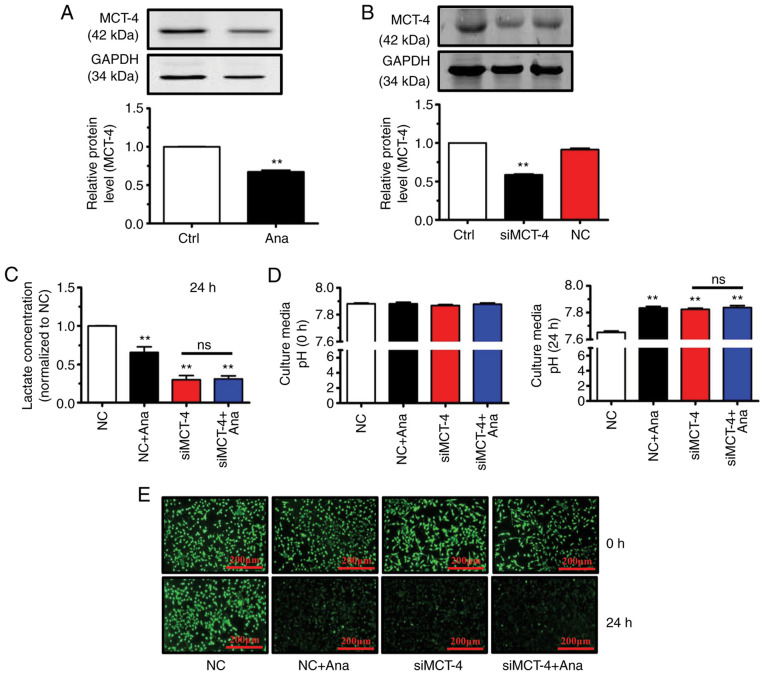
Ana suppresses MCT-4-mediated lactate excretion in CT-26 cells. (A) Treatment with Ana inhibited MCT-4 expression in CT-26 cells. ^**^P<0.01 vs. Ctrl. (B) MCT-4 siRNA suppressed MCT-4 expression in CT-26 cells. ^**^P<0.01 vs. Ctrl. (C) Ana suppressed lactate excretion in CT-26 cells after 24 h. ^**^P<0.01 vs. NC. (D) Ana reversed the low extracellular pH in CT-26 cells. ^**^P<0.01 vs. NC. (E) Ana decreased the intracellular pH in CT-26 cells (scale bars, 200 µm). The data are representative of three independent experiments performed in triplicate. Data are presented as the mean ± SD. Ana, anagliptin; MCT-4, monocarboxylate transporter-4; siRNA/si, small interfering RNA; NC, negative control; Ctrl, control; ns, not significant.

The present *in vitro* and *in vivo* experiments demonstrated that anagliptin promoted CT-26 cell apoptosis, but through an unknown mechanism. To prevent intracellular acidification, metabolic processes within cancer cells induce cytosolic accumulation of lactate and H^+^ which must be released into the extracellular space (21). A candidate protein involved in transporting lactic acid extracellularly is MCT-4(22). Anagliptin treatment decreased MCT-4 protein expression levels (Fig. 3A). It was therefore hypothesized that anagliptin affects lactate excretion via MCT-4. MCT-4 siRNA transfection efficiency in cultured CT-26 cells was therefore examined and it was determined that MCT-4 levels in these cells were reduced compared within untransfected cells (Fig. 3B).

Anagliptin-treated CT-26 cells produced lower levels of lactate in cell culture medium compared with in the negative control (NC) group (Fig. 3C). In addition, MCT-4 siRNA transfection significantly reduced lactate levels compared with in the NC group (Fig. 3C). However, co-application of MCT-4 siRNA and anagliptin produced no additive effect on lactate levels in culture medium (Fig. 3C). Since anagliptin inhibited lactate excretion in CT-26 cells, it was then assessed whether anagliptin affected lactate-induced pHe alterations. It was revealed that treatment with anagliptin reversed low pHe (Fig. 3D) while decreasing pHi levels after culturing for 24 h (Fig. 3E). The same results were obtained following MCT-4 siRNA transfection (Fig. 3D and E). However, co-application of MCT-4 siRNA and anagliptin had no further effect on the reversal of the pHi gradient (Fig. 3D and E). The results showed that treatment with anagliptin suppressed the excretion of lactate via MCT-4, then lead to the reversal of the abnormal pHi and pHe.

### Anagliptin reduces the mitochondrial membrane potential (ΔΨm) via MCT-4-mediated accumulation of lactate in CT-26 cells

Lactate strongly increases the number of reactive oxygen species in cancer cells (23). Lactate accumulation in the cytoplasm causes mitochondrial permeability, thus resulting in a reduction in ΔΨm and the induction of apoptosis (24). It was therefore hypothesized that anagliptin may induce apoptosis in CT-26 cells via MCT-4-mediated lactate accumulation.

**Figure 4:**
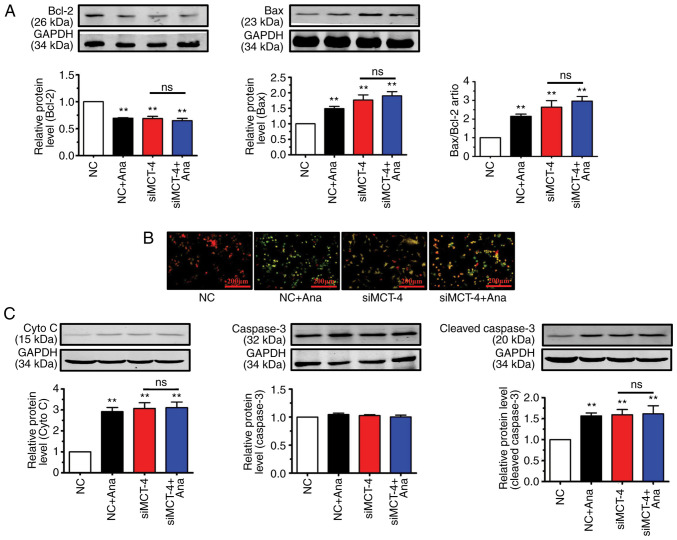
Ana promotes the expression of proteins involved in programmed cell death. (A) Western blot analysis of Bcl-2 and Bax following treatment with Ana and siMCT-4 transfection. (B) Treatment with Ana and transfection of siMCT-4 both induced the loss of ΔΨm (scale bars, 200 µm). (C) Western blot analysis of cleaved-caspase-3 and cyto C after treatment with Ana and siMCT-4 transfection. The results are representative of three independent experiments performed in triplicate. Data are presented as the mean ± SD. ^**^P<0.01 vs. NC. Ana, anagliptin; ΔΨm, mitochondrial membrane potential; NC, negative control; cytoC, cytochrome *c*; MCT-4, monocarboxylate transporter-4; si-, small interfering; ns, not significant.

Anagliptin treatment reduced Bcl-2 expression levels and increased Bax expression levels in CT-26 cells when compared with the NC group (Fig. 4A). Transfection with MCT-4 siRNA in CT-26 cells decreased the protein level of Bcl-2 and increased Bax expression compared with NC group (Fig. 4A). Co-application of anagliptin and MCT-4 siRNA produced no further effect on Bcl-2 and Bax expression. The present results showed that, after treating CT-26 cells with anagliptin, the expression of Bcl-2 was decreased and the expression of Bax was increased.

As demonstrated by JC-1 staining (representing the ΔΨm), treatment with anagliptin in CT-26 cells demonstrated a decrease in red fluorescence (red indicates aggregates with high potential) and an increase in green fluorescence (green indicates monomers, which have low ΔΨm potential, indicating lost membrane potential) in the majority of cells. Transfection of CT-26 cells with MCT-4 siRNA also led to low ΔΨm potential (decreased red fluorescence) (Fig. 4B). The same results was also observed after co-application MCT-4 siRNA and anagliptin. These results showed that treatment with anagliptin disrupted the ΔΨm potential via MCT-4.

Critical events during apoptosis are the release of cyto C from the mitochondria and caspase-3 activation (25). Anagliptin significantly increased cyto C and cleaved-caspase-3 expression in cultured CT-26 cells, but not caspase-3 (Fig. 4C). Similar results were obtained following transfection of CT-26 cells with MCT-4 siRNA (Fig. 4C). The same results were detected after co-application MCT-4 siRNA and anagliptin. But, co-application of MCT-4 siRNA and anagliptin had no further effect on cyto C and cleaved-caspase-3 expression. These results showed that treatment with anagliptin increased the expression levels of cyto C and cleaved-caspase-3, but not the expression of caspase-3.

## Discussion

In the present study, the mechanism by which anagliptin induced cellular apoptosis *in vivo* and *in vitro* was investigated, the results of which indicated that anagliptin induced apoptosis of CT-26 cells via MCT-4-mediated intracellular lactate accumulation which lead to intracellular acidosis. Antagonism of lactate shuttlingmodulatesMCT-4 expression, and is a target for predicting response to therapy. Developing pharmaceutical therapies to block this target will be a promising strategy in cancer therapy (26).

CD26/DPP4 plays an important role in several types of cancer (27-31) and DPP-4 inhibitors are being evaluated as treatments for cancer. Certain studies have indicated that anagliptin may inhibit the proliferation of tumor cells (32,33). However, in those studies, the mice were fed a diet containing a low dose of anagliptin; this was defined as ‘anagliptin mixed into the food’ (32). This method means artificial preparation of food (mixing the ingredients together), which is then fed to the animals. Mixing the active pharmaceutical ingredient with nutritional composition is simple. However, considering the physical and chemical properties of medicine, it is hard to ensure uniform distribution of medicine in food. Therefore, it must be appraised before it can be used. However, it is hard to guarantee appropriate animal intake each day. Notably, this type of administration method cannot be practically applied due to the fact that certain animals may intake markedly more than others, which may lead to the heterogeneity of treatment results. Thus, in the present study, the oral gavage method was used to guarantee uniformity. In the present study, anagliptin was used to inhibit the proliferation of tumor cells. Based on our pre-experiments, different dosages of anagliptin (10-30 mg/kg) were first applied. The results revealed that 20 and 30 mg had the same antitumor effect, but that the effect of 10 mg was weaker than that of 20 mg (Li *et al*, unpublished data). A dosage of 20 mg/kg/day anagliptin was therefore selected for use in the present study, which differs from previous studies (32,33).

The findings of the present study demonstrated that anagliptin treatment promoted CT-26 apoptosis. Cancer cells control the intracellular balance of acids and bases through mechanisms not used by normal cells, generating a non-physiological extracellular acidic microenvironment (34). Therefore, the pathological reversal of the pH gradient in the microenvironment of cancer cells is now recognized as a defining feature of these cells (35). The present data from cultured CT-26 cells indicated that the pHe value was reduced after 24 h. Anagliptin treatment reversed the pHe/pHi gradient, that is, extracellular alkalization versus intracellular acidification. These findings suggested that anagliptin contributed to the regulation of pH gradients and that the reversible regulation thereof (∆pHi/∆pHe) presents a potential therapeutic strategy against cancer.

Lactate is a metabolic byproduct of glycolysis that contributes to extracellular acidification (36). Lactate extrusion from cancer cells prevents intracellular acidification but also leads to extracellular acidosis. In the present study, the aim was to understand: i) The role of low pH in the culture medium caused by lactate excretion, and ii) how lactate excretion is essential for maintaining pHi homeostasis (37). The present data demonstrated that anagliptin inhibited lactate excretion in cultured CT-26 cells. Based on our findings, it was concluded that anagliptin reversed the pH gradient by modulating lactate release. These findings provided evidence that anagliptin may suppress lactate release, neutralize acidity in the extracellular microenvironment and decrease the pHi.

Lactate is a weak acid that cannot freely diffuse across cell membranes. MCTs are responsible for lactate release and may function as lactate exporters or importers (38). In the present study, it was found that MCT-4 was a target of anagliptin and that anagliptin treatment reduced MCT-4 protein expression levels. Notably, anagliptin prevented the excretion of lactate from CT-26 cells via MCT-4 in the present experiments after transfection of MCT-4 siRNA. Taken together, it was suggested that anagliptin may reverse the pH gradient by modulating MCT-4 expression.

In conclusion, several types of human cancer demonstrate increased MCT-4 expression, a feature reported to be associated with poor cancer prognosis (39). MCT-4 is able to secrete lactate into the microenvironment (40), which creates the ideal environment for certain acquired characteristics of cancer cells (41). The results of the present study suggested that anagliptin promoted the apoptosis of cancer cells via MCT-4-mediated lactate release. The data indicated that anagliptin reversed the abnormal pH gradient, regulating the acid-base balance. The present study observed that treatment with anagliptin had the ability to induce the apoptosis of CT-26 cells via MCT-4-mediated intracellular lactate accumulation which lead to intracellular acidosis. The function of anagliptin on the proliferation of tumor cells *in vivo* and *in vitro* was explored in the present study. However, only CT-26 cells were used to study the effect of anagliptin on apoptosis; hence, in our future studies other kinds of cancer cells will be used to detect the anti-cancer effect of anagliptin. The Na^+^/H^+^ exchanger (NHE) contributes to cellular pH homeostasis by regulating the acid-base balance; this antiporter is the predominant isoform expressed in tumors (42). Elevated NHE activity may be a major factor in promoting extracellular/interstitial acidity from the earliest stages of oncogene-driven neoplastic transformation (43). Future research should examine whether anagliptin regulates the pH gradient via NHE-mediated H^+^ excretion. It was therefore proposed that anagliptin may be a novel target for improving anticancer drug therapy.

## Data Availability

The datasets used and/or analyzed during the current study are available from the corresponding author on reasonable request.
